# Murine model of elastase-induced proximal thoracic aortic aneurysm through a midline incision in the anterior neck

**DOI:** 10.3389/fcvm.2023.953514

**Published:** 2023-02-06

**Authors:** Jianqing Deng, Dandan Li, Xuelin Zhang, Weihang Lu, Dan Rong, Xinhao Wang, Guoyi Sun, Senhao Jia, Hongpeng Zhang, Xin Jia, Wei Guo

**Affiliations:** ^1^Department of Vascular and Endovascular Surgery, The First Medical Center of PLA General Hospital, Beijing, China; ^2^State Key Laboratory of Cardiovascular Disease, Fuwai Hospital, National Center for Cardiovascular Diseases, Chinese Academy of Medical Sciences and Peking Union Medical College, Beijing, China; ^3^Department of Cardiovascular Surgery, The Sixth Medical Center of PLA General Hospital, Beijing, China

**Keywords:** thoracic aortic aneurysm, experimental models, aneurysmal pathology, elastase, aortic degenerative disease

## Abstract

**Objective:**

This study was performed to develop a murine model of elastase-induced proximal thoracic aortic aneurysms (PTAAs).

**Methods:**

The ascending thoracic aorta and aortic arch of adult C57BL/6J male mice were exposed through a midline incision in the anterior neck, followed by peri-adventitial elastase or saline application. The maximal ascending thoracic aorta diameter was measured with high-resolution micro-ultrasound. Twenty-eight days after the operation, the aortas were harvested and analyzed by histopathological examination and qualitative polymerase chain reaction to determine the basic characteristics of the aneurysmal lesions.

**Results:**

Fourteen days after the operation, the dilation rate (mean ± standard error) in the 10-min elastase application group (*n* = 10, 71.44 ± 10.45%) or 5-min application group (*n* = 9, 42.67 ± 3.72%) were significantly higher than that in the saline application group (*n* = 9, 7.37 ± 0.94%, *P* < 0.001 for both). Histopathological examination revealed aortic wall thickening, degradation of elastin fibers, loss of smooth muscle cells, more vasa vasorum, enhanced extracellular matrix degradation, augmented collagen synthesis, upregulated apoptosis and proliferation capacity of smooth muscle cells, and increased macrophages and CD4^+^ T cells infiltration in the PTAA lesions. Qualitative analyses indicated higher expression of the proinflammatory markers, matrix metalloproteinase-2 and -9 as well as Collagen III, Collagen I in the PTAAs than in the controls.

**Conclusion:**

We established a novel *in vivo* mouse model of PTAAs through a midline incision in the anterior neck by peri-adventitial application of elastase. This model may facilitate research into the pathogenesis of PTAA formation and the treatment strategy for this devastating disease.

## Introduction

An aortic aneurysm is characterized by localized permanent dilation of the aortic wall of ≥50% compared with the normal aorta. It is a silent, life-threatening disease with an increasing incidence, and it is currently the 18th most common cause of death among all individuals (1). Thoracic aortic aneurysms (TAAs) account for 35% of all aortic aneurysms, with a reported incidence of 6 to 10 per 100,000 people annually and a prevalence of 0.16 to 0.34% (2, 3). These data are probably underestimated because one study showed that 95% of cases were asymptomatic and undetected until the TAA ruptured, resulting in death (4). TAAs tend to expand in size over time, and the risk of rupture becomes markedly higher as the diameter increases (2% for TAAs ranging from 4.0 to 4.9 cm, and 7% for TAAs of > 6.0 cm) (3). TAA rupture is almost universally lethal, and prophylactic surgical repair of a TAA carries significant morbidity (5). Most patients wait about 5 years for elective surgical repair (5). This time window underscores the urgent need to develop conservative medical therapies that can stop or slow TAA enlargement and avoid surgical repair. However, no proven pharmaceutical treatments have yet been shown to effectively slow or prevent TAA progression, mainly because of the incomplete understanding of the pathogenesis of TAA (6).

The current knowledge of the pathology of TAA is mostly derived from experimental TAA models. In particular, animal models of distal descending TAA have been developed and widely used (5–12). However, the embryologic origins of the cells in the ascending aorta and aortic arch differ from those of the cells in the descending aorta (the ascending aorta and aortic arch develop from neural crest cells, whereas the descending aorta develops from the mesoderm). Different segments of the thoracic aorta develop and differentiate under different genetic and transcription factors (13). Therefore, the pathological study of TAA may be most appropriately divided into different segments. To the best of our knowledge, only a few specific models of proximal TAA (PTAA) located in the ascending aorta and aortic arch has yet been designed. Trachet et al. (14, 15) reported that angiotensin II infusion in ApoE-knockout mice could lead to ascending aortic aneurysm formation; however, most aneurysmal lesions were associated with focal dissection and intramural hematomas, which is distinct from the weakened laminated aortic wall seen in human aneurysmal disease (14, 15). This model also led to abdominal dissecting aneurysms and was reported to be a more clinically relevant model of aortic dissection (16). Radu et al. (17) exposed rat ascending aorta *via* median sternotomy, followed by 40-min peri-aortic application of elastase to induce ascending aortic aneurysm. This PTAA model involves opening the thoracic cavity, which requires positive-pressure ventilation. Therefore, we established a novel method to induce PTAA located in the ascending aorta and aortic arch by peri-adventitial application of elastase through a midline incision in the anterior neck, avoiding thoracotomy and tracheal intubation. This minimally invasive method produces reproducible, robust aortic dilation in the ascending aorta and aortic arch.

## Materials and methods

All animal experimental protocols were reviewed and approved by the Animal Care and Use Committee, Experimental Animal Center, Fuwai Hospital, National Center for Cardiovascular Diseases, China (FW-2021-0023).

### Surgical procedure and follow-up

**FIGURE 1:**
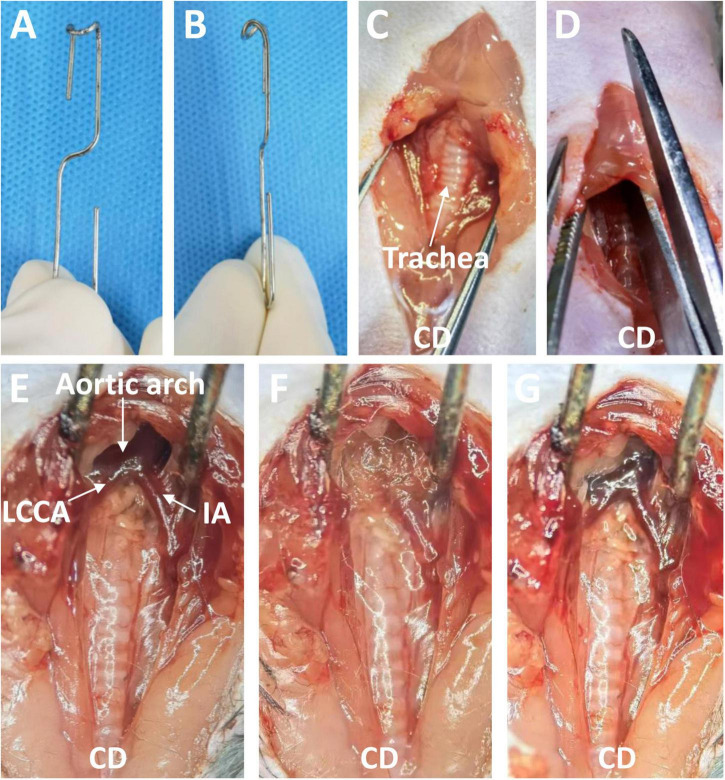
The blunt hook and the surgical process used to induce a thoracic aortic aneurysm through a midline incision in the anterior neck. **(A)** Front view of the blunt hook. **(B)** Side view of the blunt hook. **(C)** The skin was opened, and the thyroid gland and muscles in front of the trachea were pulled toward both sides. **(D)** The sternum was cut to the second rib. **(E)** The sternum was pulled 30 to 45 degrees forward and upward, exposing the targeted aorta. **(F)** An elastase- or saline-soaked sponge was applied. **(G)** The sponge was removed (Elastase application group). CD, cephalad direction; IA, innominate artery; LCCA, left common cervical artery.

Eight- to 12-week-old male C57BL/6J mice were anesthetized by intraperitoneal injection of 2% tribromoethanol (350 mg/kg). Depilatory cream was used to remove the hair on the neck and front chest. Next, each mouse was placed in the supine position under a stereoscope. The operator sat cephalad to the mouse. After disinfection of the surgical area, the operator opened the skin from the neck to the second intercostal space of the thorax along the anterior midline with scissors, then pulled the thyroid gland and muscles in front of the trachea toward both sides with blunt separating forceps. Next, the sternum was cut to the second rib (approximately 5 mm), and the thymus lobes and sternum were pulled 30 to 45 degrees forward and upward with a blunt hook (Figures 1A, B). At this point, the distal ascending aorta, transverse aortic arch, innominate artery, and left common cervical artery were visible. The aforementioned surgical procedure is similar to the procedure used for minimally invasive transverse aortic constriction by Zaw et al. (18). The connective tissues and adipose tissues around the entire ascending and arch were carefully removed with microsurgical forceps, so that the topical elastase can reach the sides and back of the aorta. A sponge measuring about 5 × 1 × 1 mm soaked with 15 μL of porcine elastase (E1250; Type I, 5.0 units/mg protein, 12 mg protein/mL, Sigma-Aldrich, St. Louis, MO, USA) or 0.9% saline was placed on the exposed thoracic aorta for 5 or 10 min. Finally, the sponge was removed, the surgical site was rinsed three times with saline solution, and the chest and surgical incision were closed. Five percent lidocaine cream was applied to the surgical wound to alleviate pain. The main surgical procedures are briefly shown in Figures 1C–G. The mice were allowed to recover for several minutes on an insulation blanket. After full recovery, the mice were returned to a 12-h light/dark cycle room in specific pathogen-free animal laboratory and fed standard chow and water.

In this study, the maximal diameter of the ascending thoracic aorta represented the size of the PTAA or the saline-treated thoracic aorta. The maximal diameter of the ascending thoracic aorta was measured before and after the operation with a high-resolution micro-ultrasound system (Vevo 2100; FUJIFILM VisualSonics Inc., Toronto, ON, Canada) equipped with a MS400 transducer (18 to 38 MHz). For ultrasonic scanning, the hair on the front chest of mice was removed and the mice were anesthetized with 1% isoflurane. In the supine position, the probe was placed on the right edge of the mouse sternum. The probe formed a 45 degrees angle with the chest wall. The images of ascending aorta, aortic arch and its branches were obtained. Longitudinal images of the aorta were obtained in B mode and the inner diameter of the largest ascending aorta was measured.

### Histopathological examination

The mice with over 50% thoracic aorta expansion in aortic ultrasound examination were euthanized 28 days after the operation. The thoracic cavity was opened and the right atrial appendage was cut out. Next, 10 mL of phosphate-buffered saline and 5 mL of 4% paraformaldehyde solution were successively injected into the apex of the heart. The adipose tissue around the thoracic aorta was then removed under a stereoscope. The harvested thoracic aortas were fixed in 4% paraformaldehyde for 24 h. Dehydration and paraffin embedding were then successively carried out. Finally, the aortic tissues were sliced into 3-μm-thick serial sections. The sections underwent hematoxylin and eosin staining and Elastic Van Gieson staining (ab150667; Abcam, Cambridge, UK), after which they were captured with a Pannoramic SCAN II system (3DHISTECH Ltd., Budapest, Hungary). Elastin fragmentation was graded as Yang et al. (19) did, detailed fragmentation scoring rules were provided in the legends of Supplementary Figure 1.

After routine dewaxing and hydration, the antigens were retrieved by a heat-mediated method. For immunohistochemistry, the sections were pretreated using endogenous peroxidase blocking solution for 30 min, then blocked with 3% bovine serum albumin/10% normal goat serum. The primary antibodies with distinct dilution ratio was incubated with the sections at 4°C for 16 h. The primary antibodies and the dilution ratio was provided in the shown in Supplementary Table 1. Next, a goat horseradish peroxidase-conjugated anti-rabbit or mouse secondary antibody solution was incubated with the sections for 1 h at 37°C; 3,3N-diaminobenzidine tetrahydrochloride was used as the chromogen. This was followed by hematoxylin staining, neutral balata fixation, and capturing with the Pannoramic SCAN II system (3DHISTECH Ltd., Budapest, Hungary). The Image Pro Plus software (Version 6.0.0.260, Media Cybernetics, Inc., Rockville, MD, USA) was used to quantify immunohistochemistry images.

For immunofluorescence, the sections were permeabilized with phosphate-buffered saline solution containing 0.1% Triton X-100 for 15 min after antigen retrieval. Next, the sections were blocked with 3% bovine serum albumin/10% normal goat serum and incubated with primary antibodies with distinct dilution ratio at 4°C (Supplementary Table 1). Sixteen hours later, Alexa Fluor 488/594 goat anti-rabbit/mouse IgG antibody with an appropriate dilution ratio were incubated with the sections for 1 h in the dark at 37°C, followed by DAPI nuclear staining. We observed the sections and captured the images of interest with a Leica SP8 laser scanning confocal microscope (Leica, Wetzlar, Germany).

### Reverse-transcription polymerase chain reaction (RT-PCR)

Twenty-eight days after the operation, the murine PTAAs or saline-treated thoracic aortas were harvested and placed in liquid nitrogen. Next, snap-frozen tissue samples were ground into powder using a mortar. A proper volume TRIzol Reagent (Invitrogen, Carlsbad, CA, USA) was then added to the mortar, and total RNA was extracted. A total of 300 ng of total RNA was reversed to cDNA using an iScript cDNA Synthesis kit (#1708891; Bio-Rad Laboratories, Hercules, CA, USA) in the ProFlex Base PCR System (Applied Biosystems, Thermo Fisher Scientific, Waltham, MA, USA). Real-time PCR was performed using iTaq Universal SYBR Green Supermix (#1755121; Bio-Rad Laboratories, Hercules, CA, USA) in the QuantStudio 6 Flex Real-Time PCR System (Applied Biosystems, Thermo Fisher Scientific, Waltham, MA, USA) with the primers shown in Supplementary Table 2.

### Western blot analysis

Immunoblot analysis of the Collagen III, Collagen I, matrix metalloproteinase (MMP) 2 and MMP9 were performed using protein extraction from sham-operated thoracic aorta and the PTAAs (28 days after surgery). NuPAGE 4–12% Bis-Tris gel (Thermofisher, NP0335BOX), NuPAGE MES SDS running buffer (Thermofisher, NP0002) were used for protein electrophoresis. 30 μg denatured protein was added into the lane of 4–12% Bis-Tris gel (1.0–1.5 mm, 10 wells). The protein electrophoresis was performed at 110V for 1.5 h. Then, the mini-size stack (8 cm x 8 cm, Thermofisher, IB24002), an integrated pre-activated PVDF transfer membrane was used for dry protein transferring, which was finished in 7 min by using the iBlot 2 Gel Transfer Device. QuickBlock™ Blocking Buffer (Beyotime, P0252) was used for the blocking step (25°C, 1 h). Afterward, the membrane was incubated in primary antibodies with distinct dilution ratio (Supplementary Table 1) at 4°C for 16 h, followed by incubation with distinct secondary antibody solution (Supplementary Table 1) at room temperature for 1 h. Finally, images were acquired using a Tanon automatic chemiluminescence imaging system and the optical density of protein bands were determined by Image J pro plus software (Version 6.0.0.260, Media Cybernetics, Inc., Rockville, MD, USA).

### Statistical analysis

All statistical analyses were performed using GraphPad Prism 9.0 (GraphPad Software, San Diego, CA, USA). All qualitative results are expressed as mean ± standard error. Two tailed Mann–Whitney *U* test was used for comparison of two groups.

## Results

### Dilation of proximal thoracic aorta after peri-adventitial application of saline or elastase

**FIGURE 2:**
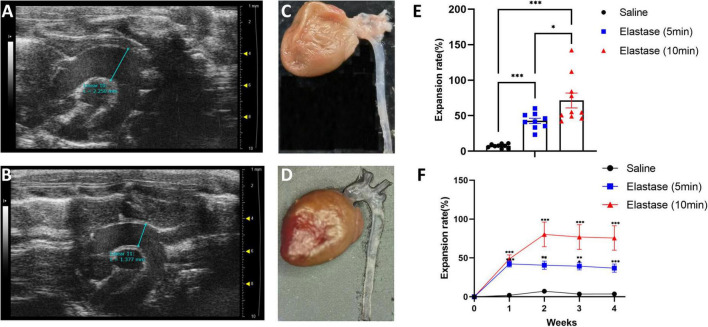
The ultrasonic image, morphology, and the expansion rate of proximal thoracic aortic aneurysm (PTAA) models. **(A)** The representative ultrasonic image of the PTAA (5-min elastase application), the maximal ascending aorta diameter is 2.25 mm 28 days after operation. **(B)** The representative ultrasonic image of the controlled thoracic aorta, the maximal ascending diameter is 1.38 mm 28 days after operation. **(C)** Representative morphology of PTAA. **(D)** Representative morphology of the controlled thoracic aorta. **(E)** Fourteen days after operation, the aortic dilation rate in 5-min, 10-min elastase, and saline application groups. The aortic dilation rate in different groups were compared with the two-tailed Mann–Whitney tests,*P < 0.05, ^***^P < 0.001. **(F)** Aortic dilation rate over time during PTAA formation. Maximal ascending aortic diameter (mean ± s.e.m.) in male C57BL/6J exposed to 5-min (*n* = 9) or 10-min (*n* = 9) elastase application or exposed to 10-min saline (*n* = 9) over time. The aortic dilation rate between the 5-min or 10-min elastase application group and the saline group were compared with the two-tailed Mann–Whitney tests, ^**^P < 0.01, ^***^P < 0.001.

Totally, 29 mice underwent the operation (9 in the saline application group, 9 in the 5-min elastase application group, and 11 in the 10-min elastase application group). The operative time (except elastase or saline application time) per mouse was 16.89 ± 0.47 min (mean ± standard error). Two mice in the 10-min group died of thoracic aortic aneurysm rupture on the seventh and eighteenth day after operation. Their maximal diameter of thoracic aortic aneurysm reached 2.78 and 2.01 mm in the last ultrasonic examination, respectively. Peri-adventitial application of elastase to the proximal thoracic aorta led to significant aortic dilation (Figures 2A, C) compared with peri-adventitial application of saline (Figures 2B, D). As is shown in Figure 2E, fourteen days after the operation, the dilation rate (mean ± standard error) in the 10-min elastase application group (*n* = 10, 71.44 ± 10.45%) and 5-min application group (*n* = 9, 42.67 ± 3.72%) were significantly higher than that in the saline application group (*n* = 9, 7.37 ± 0.94%, all *P* < 0.001). Ten-min elastase application led to significantly larger aortic dilation than 5-min elastase application (Figure 2E, *P* < 0.05). The mean aortic size was 1.43 ± 0.02 mm in the control group, 2.01 ± 0.05 mm in the 5-min application group, and 2.37 ± 0.15 mm in the 10-min application group. To show the trend of aortic dilatation over time, the aortic diameter data of mice that underwent preoperative and weekly ultrasound after the operation are summarized in Figure 2F. In the 5-min application group, the maximal aortic expansion occurred on postoperative day 7 (42.23 ± 3.92%), and a slight trend of regression in size was observed on day 28 (36.94 ± 5.30%). In the 10-min application group, the maximal aortic dilation occurred on postoperative day 14 (80.36 ± 15.97%), and a slight trend of aortic regression in size was also observed on day 28 (75.71 ± 15.73%).

### Histopathological examination

#### The basic characteristics of aortic wall structure and contents

**FIGURE 3:**
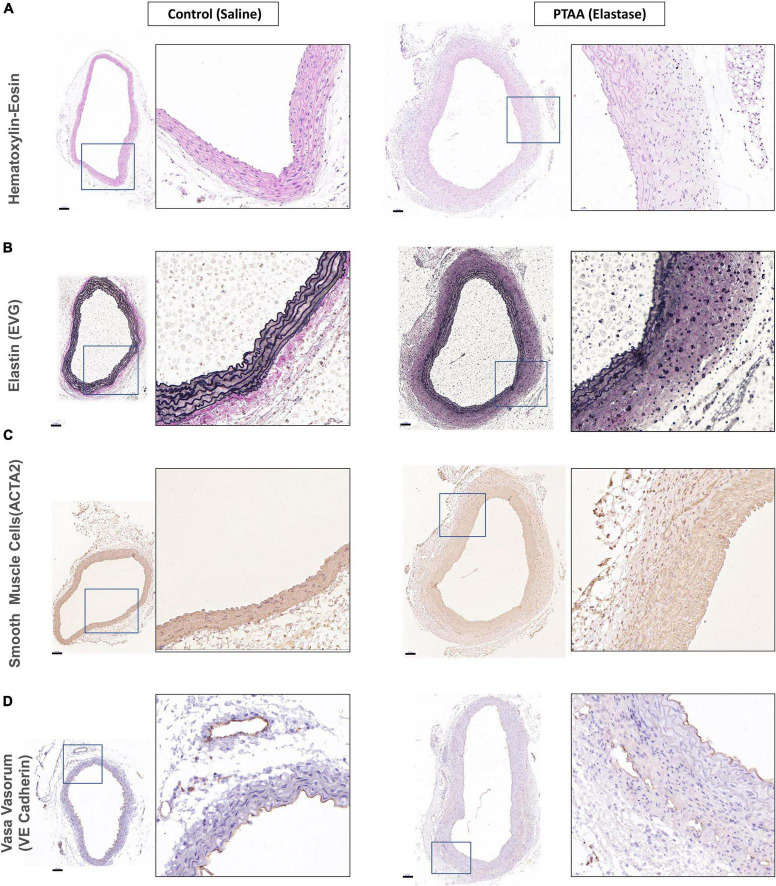
Histopathological examination of ascending aorta in control (saline) and proximal thoracic aortic aneurysm (PTAA) (Elastase). **(A)** Representative images of hematoxylin-eosin staining in both groups. **(B)** Representative images of elastin (EVG) staining in both groups. **(C)** Representative images of ACTA2 immunohistochemical staining (smooth muscle cells) in both groups. **(D)** Representative images of VE-Cadherin immunohistochemical staining (vasa vasorum) in both groups. Scale bar in the left figure showed is 100 μm, the magnification of the local enlarged view on the right is 6.25.

All three mice in the 5-min group with over 50% thoracic aorta expansion and two randomly selected mice in the 10-min group with over 50% thoracic aorta expansion were used for histopathological examination. As shown in Figure 3A, the PTAAs had an obviously larger lumen and adventitia thickness than the saline-treated aortas. The aorta wall thickness and cross-sectional area of the PTAAs were significantly higher than that in the saline-treated aorta (Supplementary Figures 1A, B, *P* < 0.05 for both). Elastic Van Gieson staining showed that the elastin fibers of the PTAA were degraded but that they were preserved and intense in the saline-treated aorta, which is confirmed by the significantly higher elastin fragmentation score in PTAAs (Figure 3B and Supplementary Figure 1C, *P* < 0.05). Immunostaining for smooth muscle cell (SMC) with ACTA2 antibody showed a greater ACTA2-negative area in the media of the PTAA, and the mean optical density of ACTA2 was significantly lower in PTAA lesions than in the control aortas (Figure 3C and Supplementary Figure 1D, *P* < 0.05), indicating SMC loss. Significantly more vasa vasorum were observed in the PTAA lesions, mainly located in the adventitia, through immunostaining for vasa vasorum with vascular endothelial (VE)-cadherin antibody (Figure 3D, Supplementary Figure 1E, *P* < 0.05).

### The apoptosis and proliferation capacity of SMCs in PTAAs

**FIGURE 4:**
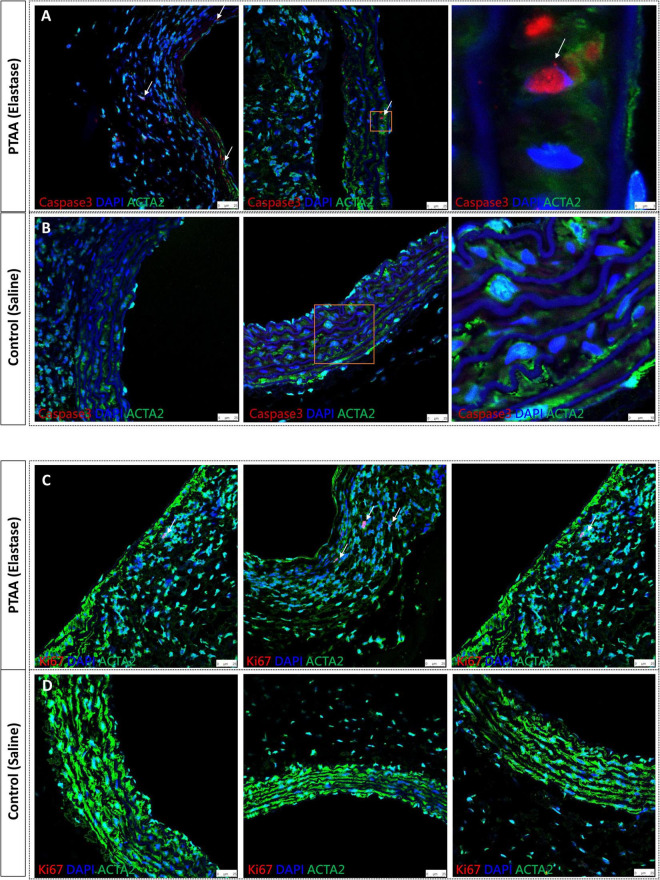
Activated caspase 3 and Ki67 immunofluorescence staining of proximal thoracic aortic aneurysms (PTAAs). **(A)** Representative immunofluorescence images of activated caspase 3 (Red), ACTA2 (Green) and nuclear (DAPI, Blue) in the PTAA sections and **(B)** in the control aortas. White arrows indicate the caspase 3 positive smooth muscle cells (SMCs). **(C)** Representative immunofluorescence images of Ki67 (Red), ACTA2 (Green) and nuclear (DAPI, Blue) in the PTAA sections and **(D)** in the control aortas. White arrows indicate the Ki67 positive SMCs.

Activated caspase-3 is derived from cleavage of procaspase-3 by an initiator caspase induced by apoptotic signaling (15). Figures 4A, B shows activated caspase-3 positive, in other words, apoptotic SMCs were present in PTAAs, whereas no apoptotic SMCs were found in the saline-treated aortas. Furthermore, the Ki67 nuclear protein is present during the active phases of the cell cycle, so it is often used as a proliferation marker (20). The immunofluorescence of Ki67 indicated that Ki67 positive SMCs was only present in the PTAA tissue. The typical positive findings are shown in Figures 4C, D.

### The expression of proinflammatory markers, MMP2, MMP9, Collagen III, Collagen I in PTAAs

**FIGURE 5:**
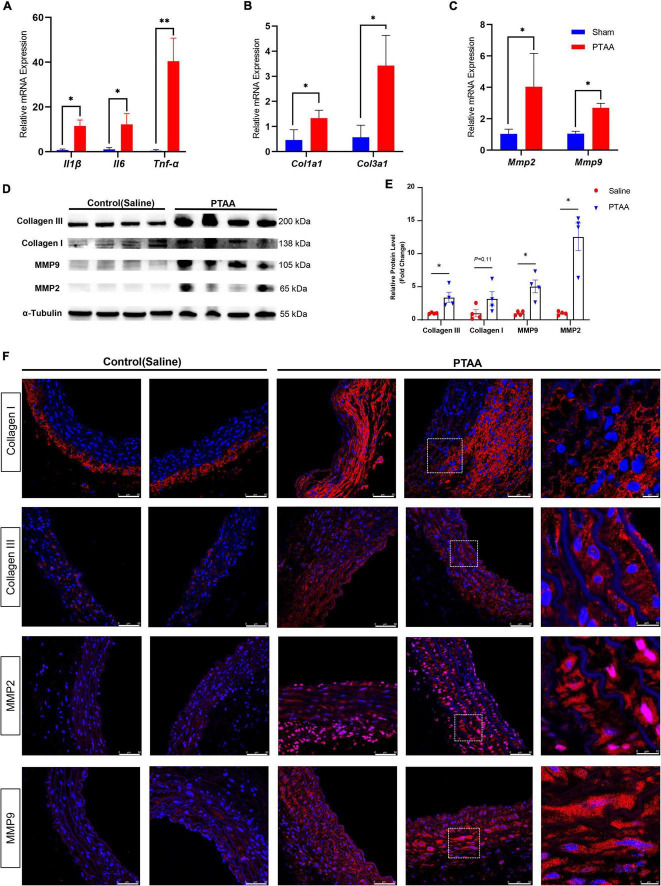
Quantification analysis and immunofluorescence staining of the matrix metalloproteinase and collagen molecules. **(A)** RT-qPCR analysis of the proinflammatory markers, *IL-1*β, *IL-6, TNF*-α. **(B)** RT-qPCR analysis of the *Col1a1, Col3a1*. **(C)** RT-qPCR analysis of the *Mmp2* and *Mmp9*; *n* = 4 in both groups, the difference was compared by two-tailed Mann–Whitney tests, *P < 0.05, ^**^P < 0.01. **(D)** Immunoblot analysis of the COL1A1, COL3A1, MMP2, and MMP9 in protein lysates from sham-operated thoracic aorta and the PTAAs (28 days after surgery). **(E)** Quantification analysis of the protein density in the forementioned Immunoblot analysis. Mean ± s.e.m.; by two-tailed Mann–Whitney tests; *P < 0.05. **(F)** Representative immunofluorescence images of proximal thoracic aortic aneurysm (PTAA) lesions or controlled thoracic aorta stained for COL1A1, COL3A1, MMP2 or MMP9 (Red), and DAPI (blue).

Quantitative RT-qPCR analysis indicated that several proinflammatory markers, including tumor necrosis factor-α, interleukin-1β, and interleukin-6, were significantly increased in PTAA tissues (Figure 5A, all *P* < 0.05). Similarly, the mRNA levels of *Col1a1*, *Col3a1*, *Mmp2*, and *Mmp9* were significantly elevated (Figures 5B, C, *P* < 0.05 for all). Western blot results showed that the protein levels of Collagen III, Collagen I, MMP2, and MMP9 were also upregulated in the PTAA lesions (Figure 5D), quantitative gray-scale analysis showed that these genes had statistically significant elevation in protein levels except Collagen I, which only showed an increasing trend (Figure 5E, *P* < 0.05 for Collagen III, MMP2, and MMP9, *P* = 0.11 for Collagen I), indicating upregulated collagen synthesis and enhanced extracellular matrix degradation in PTAA tissues. The typical results of immunofluorescence in Figure 5F showed Collagen III, Collagen I, MMP2, and MMP9 located at the adventitia and interlamellar spaces in the PTAA lesions. In the control group, Collagen III, MMP2, and MMP9 mainly located in the aortic medial layer, whereas Collagen I located at the adventitia predominantly.

### Aortic immune cell infiltration in PTAAs

**FIGURE 6:**
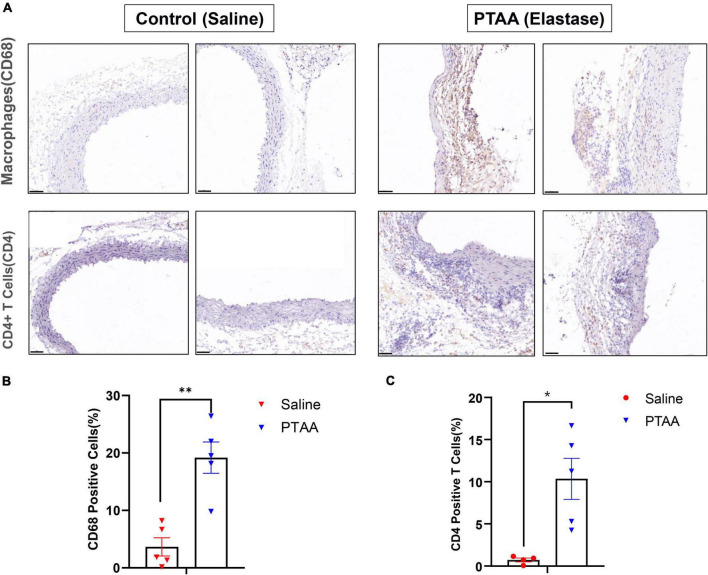
Histopathological examination of Macrophages and CD4^+^ T cells in control (saline) and proximal thoracic aortic aneurysm (PTAA) (Elastase) tissue. **(A)** Representative immunohistochemistry images of CD68 and CD4 staining in both groups. Scale bar in the bottom left corner is 50 μm. **(B)** Quantification of the CD68 positive macrophages in both groups. **(C)** Quantification of the CD4^+^ T Cells in both groups. Mean ± s.e.m; two-tailed Mann–Whitney tests, *P < 0.05 and **P < 0.01.

Macrophages and CD4^+^ T cells, which dominate among the inflammatory mononuclear cells and play an important role in aneurysmal formation and progression, are abundant infiltrates in aneurysmal lesions (21–23). We quantified the expression of CD68 (a biomarker of macrophage) and CD4 (a biomarker of CD4^+^ T cell) to assess macrophage and T cell infiltration in PTAAs by immunohistochemistry staining. Compared with the sham aorta, greater infiltration of CD68 positive macrophages was observed in the PTAA lesions, especially in the adventitia of the aneurysm (Figures 6A, B, *P* < 0.01). Similarly, we also found a significantly more CD4^+^ T cells in the adventitia of PTAA lesions (Figures 6A, C, *P* < 0.05).

## Discussion

In this study, we established a model of PTAA through a midline incision in the anterior neck by peri-aortic application of elastase. The PTAA lesions exhibited wall thickening, degradation of elastic fibers, loss of SMCs, more vasa vasorum, enhanced extracellular matrix degradation, augmented collagen synthesis, upregulated apoptosis and proliferation capacity of SMCs, and greater macrophages and CD4^+^ T cells infiltration, which are mostly in line with the basic pathology of aneurysmal lesions in other experimental models and human aneurysmal studies (12, 14, 15, 23, 24). Therefore, our newly established experimental PTAA model could facilitate research into the key processes that play a role in PTAA formation and the treatment strategy for this devastating disease.

Notably, there were more newly formed vasa vasorum in the PTAA lesions, which is consistent with the Zollikofer et al’ s study (25), this can be explained by the fact that the vasa vasorum proliferate to meet the increased oxygen and nutrition demand caused by aortic wall thickening and healing. Greater macrophages infiltration was found in the PTAA lesions, macrophages produce excessive MMPs that cause degradation of extracellular matrix and thereby weaken the strength of the aortic wall (23). We have also found that much more CD4^+^ T cells infiltrate in the PTAA lesions. CD4^+^ T cells and interferon-γ(a prototypical cytokine of Th1 cells, a subgroup of CD4^+^ T cells) has been demonstrated to stimulate MMP2 and MMP9 production from macrophages and SMCs, leading to structural extracellular matrix degradation and aortic dilation in murine calcium chloride (CaCl_2_)-induced aneurysm model (21). Positive correlation between IFN-γ-producing T cells (Th1) and increasing aortic diameter in clinical specimens of ascending TAA has been reported, demonstrating the important role of CD4^+^ T cells in ascending TAA formation (26).

Several experimental TAA models have been established during last decades. Radu et al. (17) exposed rat ascending aorta *via* median sternotomy, followed by 40-min peri-aortic application of elastase to induce ascending aortic aneurysm. A peri-aortic elastase murine descending TAA model was also developed by thoracic aortic exposure through a left thoracotomy incision (5, 6). Compared with these models, our experimental PTAA model is less technically challenging to establish and causes less surgical trauma. The aforementioned TAA models involve opening the thoracic cavity, which requires positive-pressure ventilation. Orotracheal intubation is relatively difficult to master, and reintubation after dislodgment of a successfully placed tube can increase the risk of fatal airway injury. Additionally, the extremely delicate mouse lung is easily injured by puncture, inappropriate handling, or elastase exposure, always resulting in death. Avoidance of lung injury requires surgical skill and experience (5). The operation used to establish our model avoids opening the thoracic cavity and orotracheal intubation; instead, we expose the proximal thoracic aorta more easily through a midline incision in the anterior neck. During the operation, we advise paying close attention to avoid injuring the proximal thoracic aorta when removing the connective or adipose tissues around the aorta with microsurgical forceps. Furthermore, because the digestive effect of elastase varies from bottle to bottle (5), the appropriate volume and application time should be determined before the formal operation to ensure sufficient digestion leading to maximal dilation while simultaneously avoiding excessive elastase digestion, which would result in intraoperative bleeding or premature rupture.

Compared with the descending TAA model developed by Tyerman et al. (5), much lower aortic regression rate in 28th day in our PTAA model was observed (about 5% in PTAA and more than 40% in descending TAA model). The rapid hemodynamic force change and higher blood flow shear stress in the proximal thoracic aortic region may hinder the aortic regression caused by aortic wall healing. The differences in embryological origins between SMCs or aortic segments could contribute to site-specific aortic aneurysm pathogenesis (27, 28). The specific detailed differences between murine PTAA lesions and descending TAA lesions at the molecular level need to be revealed through genome wide RNA sequencing in the future.

Physiologically, peri-aortic degradation by elastase results in adventitial inflammatory homing with a resultant increased inflammatory state and higher expression of MMP2 and MMP9 in the media. These changes lead to extracellular matrix degradation and SMC loss and finally to aortic weakness and dilation. This is partly in line with the prevailing theory that aneurysms develop secondary to inflammatory infiltration that initiates in the adventitia (15). Elastase perfusion or peri-aortic application of CaCl_2_ is also used to induce aneurysm formation (29). In this experimental model of PTAA, we utilized peri-adventitial application of elastase rather than elastase intraluminal perfusion because elastase perfusion into the proximal thoracic aorta is much more technically challenging, and blocking the blood supply of the cervical artery can cause cerebral ischemia, stroke, and even brain death. Additionally, we did not adopt peri-aortic application of CaCl_2_ because peri-aortic application of CaCl_2_ during the operation is time-consuming (15 min for CaCl_2_ and ≤ 10 min for elastase). CaCl_2_ also produces a much lower percent dilation in a longer period of time after surgery (25% dilation at 28 days for CaCl_2_ versus 100–130% dilation at 14 days for elastase in the descending thoracic aorta) (5, 9).

Our model has two main limitations. First, our model could not completely reproduce the human PTAA pathology. PTAA occurs in 7 or 14 days after peri-aortic application of elastase in our model, whereas human PTAA formation is always multifactorial and occurs over a period of years. However, an experimental model that takes years to develop aneurysms would be ineffective for research purposes. Second, unlike human aneurysmal disease, the elastase-induced PTAAs in our model begin to decrease in size when they reach maximal dilatation postoperatively, indicating limited potential for examining the long-term effect of tested drugs on established PTAAs. This deficiency may be overcome with the additional postoperative use of β-aminopropionitrile (BAPN), an inhibitor of lysyl oxidase (an enzyme that crosslinks elastin and collagen, maintaining the elastic lamellar structure), or systemic blockade of transforming growth factor-β (TGFβ) (a guardian of vascular integrity and immune homeostasis) (30, 31). When combined with the elastase exposure model, BAPN supplementation and systemic blockade of TGFβ allow sustained aneurysmal growth, the formation of an intraluminal thrombus, and eventual spontaneous rupture, better mimicking the major features of human disease (30, 31). The aforementioned chronic experimental model involved abdominal aneurysms. Construction of a chronic PTAA model by combining the elastase exposure model with BAPN supplementation or systemic blockade of TGFβ is needed because no such model has been developed for investigation of TAA. However, we have concerns that BAPN supplementation may result in aortic dissection in the PTAA because BAPN has the capacity to induce thoracic aortic dissection (32); moreover, the rapid hemodynamic changes and blood flow shear force may also lead to aortic dissection in the PTAA. Nevertheless, these two methods are worth trying. It would also be meaningful to develop a novel aortic dissection experimental model because ascending aorta dilation is an established morphological risk factor for Stanford type A aortic dissection (33).

## Conclusion

We established a novel *in vivo* murine model of PTAAs by peri-adventitial application of elastase through a midline incision in the anterior neck. This model may facilitate research into the pathogenesis of PTAA formation and the treatment strategy for this devastating disease.

## Data availability statement

The raw data supporting the conclusions of this article will be made available by the authors, without undue reservation.

## Ethics statement

The animal study was reviewed and approved by the Animal Care and Use Committee, Experimental Animal Center, Fuwai Hospital, National Center for Cardiovascular Diseases, China.

## Author contributions

WG supervised the study. WG, JD, and DL designed the study. JD, DL, WL, DR, GS, and SJ carried out the animal experiments. JD, DL, and XZ performed the high-resolution micro-ultrasound examination. XZ and XW carried out the histopathological experiments. JD performed the Western Blot and RT-qPCR experiments, WG, HZ, and XJ analyzed and interpreted the results. JD drafted the manuscript. All the authors revised and approved this manuscript.

## Supplementary material

Supplementary Figure 1 Statistical quantative analysis of aortic wall thickness **(A)**, cross-sectional area **(B)**, the elastin fragmentation score **(C)**, ACTA2 protein density **(D)**, and the vasa vasorum counts **(E)** of the ascending aorta in the elastase application group and the saline group. Mean ± s.e.m; two-tailed Mann–Whitney tests, **P* < 0.05. Elastin fragmentation was graded as follows: grade 1, intact, well-organized elastic laminae; grade 2, elastic laminae with some interruptions and breaks; grade 3, severe elastin fragmentation or loss; and grade 4, severe elastin degradation with visible ruptured sites.
