# Supplementary material for: Murine model of elastase-induced proximal thoracic aortic aneurysm through a midline incision in the anterior neck
Source: Front Cardiovasc Med. 2023 Feb 6;10:953514. doi: 10.3389/fcvm.2023.953514 (PMC9939838; doi:10.3389/fcvm.2023.953514)
Supplement: Supplementary file 1 [file Table_1.DOCX]

**Supplemental Table I. The antibodies list for western blot,** **immunofluorescence, and immunohistochemistry**

| Antibody Name | Company | Catalog Number | Source | Applications | Dulition Ratio |
| --- | --- | --- | --- | --- | --- |
| Collagen I | Abcam | ab21286 | Rabbit | Western Blot | 1:500 |
| Collagen III | Abcam | ab184993 | Rabbit | Western Blot | 1:1000 |
| MMP2 | Abcam | ab92536 | Rabbit | Western Blot | 1:1000 |
| MMP9 | Abcam | ab38898 | Rabbit | Western Blot | 1:1000 |
| α-Tubulin | Beyotime | AF0001 | Rabbit | Western Blot | 1:1000 |
| Anti-Rabbit IgG HRP-linked Antibody | Cell Signaling Technology | 7074S | Goat | Western Blot | 1:2000 |
| COL1A1(Alexa Fluor® 647 Conjugate) | Cell Signaling Technology | 72827s | Rabbit | Immunofluorescence | 1:20 |
| Collagen III | Abcam | ab184993 | Rabbit | Immunofluorescence | 1:50 |
| MMP2 | Sigma | SAB2108458 | Rabbit | Immunofluorescence | 1:100 |
| MMP9 | Abcam | ab76003 | Rabbit | Immunofluorescence | 1:50 |
| ACTA2 | Sigma | A5228 | Mouse | Immunofluorescence | 1:500 |
| Ki67 | Abcam | ab16667 | Rabbit | Immunofluorescence | 1:200 |
| Cleaved Caspase-3 | Cell signaling Technology | 9661 | Rabbit | Immunofluorescence | 1:100 |
| Goat Anti-Rabbit IgG H&L  (Alexa Fluor® 594) | Abcam | ab150080 | Goat | Immunofluorescence | 1:500 |
| Goat Anti-Mouse IgG H&L  (Alexa Fluor® 488) | Abcam | ab150113 | Goat | Immunofluorescence | 1:500 |
| ACTA2 | Sigma | A5228 | Mouse | Immunohistochemistry | 1:500 |
| VE Cadherin | Abcam | ab205336 | Rabbit | Immunohistochemistry | 1:100 |
| CD68 | Abcam | ab955 | Mouse | Immunohistochemistry | 1:1000 |
| CD4 | Abcam | ab183685 | Rabbit | Immunohistochemistry | 1:1000 |
| HRP-labeled Goat Anti-Rabbit IgG(H+L) | ZSGB-Bio | PV-6000 | Goat | Immunohistochemistry | 1:1 |
| HRP-labeled Goat Anti-Mouse IgG(H+L) | ZSGB-Bio | PV-6000 | Goat | Immunohistochemistry | 1:1 |
