# Supplementary material for: Murine model of elastase-induced proximal thoracic aortic aneurysm through a midline incision in the anterior neck
Source: Front Cardiovasc Med. 2023 Feb 6;10:953514. doi: 10.3389/fcvm.2023.953514 (PMC9939838; doi:10.3389/fcvm.2023.953514)
Supplement: Supplementary file 2 [file Table_2.DOCX]

**Supplemental Table II.Quantitative real-time PCR primers sequence**

| Primer name | Sequence(5’-3’) |
| --- | --- |
| Il1β-F | AAATGCCACCTTTTGACAGTGAT |
| Il1β-R | AATGGGAACGTCACACACCA |
| Il6-F | CTCCCAACAGACCTGTCTATAC |
| Il6-R | CCATTGCACAACTCTTTTCTCA |
| Tnfα-F | GGTGCCTATGTCTCAGCCTCTT |
| Tnfα-R | GCCATAGAACTGATGAGAGGGAG |
| Col1a1-F | GCTCCTCTTAGGGGCCACT |
| Col1a1-R | ATTGGGGACCCTTAGGCCAT |
| Col3a1-F | GACCAAAAGGTGATGCTGGACAG |
| Col3a1-R | CAAGACCTCGTGCTCCAGTTAG |
| [Mmp2-F](https://www.ncbi.nlm.nih.gov/gene/17390) | ACCTGAACACTTTCTATGGCTG |
| [Mmp2-R](https://www.ncbi.nlm.nih.gov/gene/17390) | CTTCCGCATGGTCTCGATG |
| Mmp9-F | GCTGACTACGATAAGGACGGCA |
| Mmp9-R | TAGTGGTGCAGGCAGAGTAGGA |
| Gapdh-F | CCCCAATGTGTCCGTCGTG |
| Gapdh-R | TGCCTGCTTCACCACCTTCT |
