# Supplementary figures and images for: Murine model of elastase-induced proximal thoracic aortic aneurysm through a midline incision in the anterior neck
Source: Front Cardiovasc Med. 2023 Feb 6;10:953514. doi: 10.3389/fcvm.2023.953514 (PMC9939838; doi:10.3389/fcvm.2023.953514)

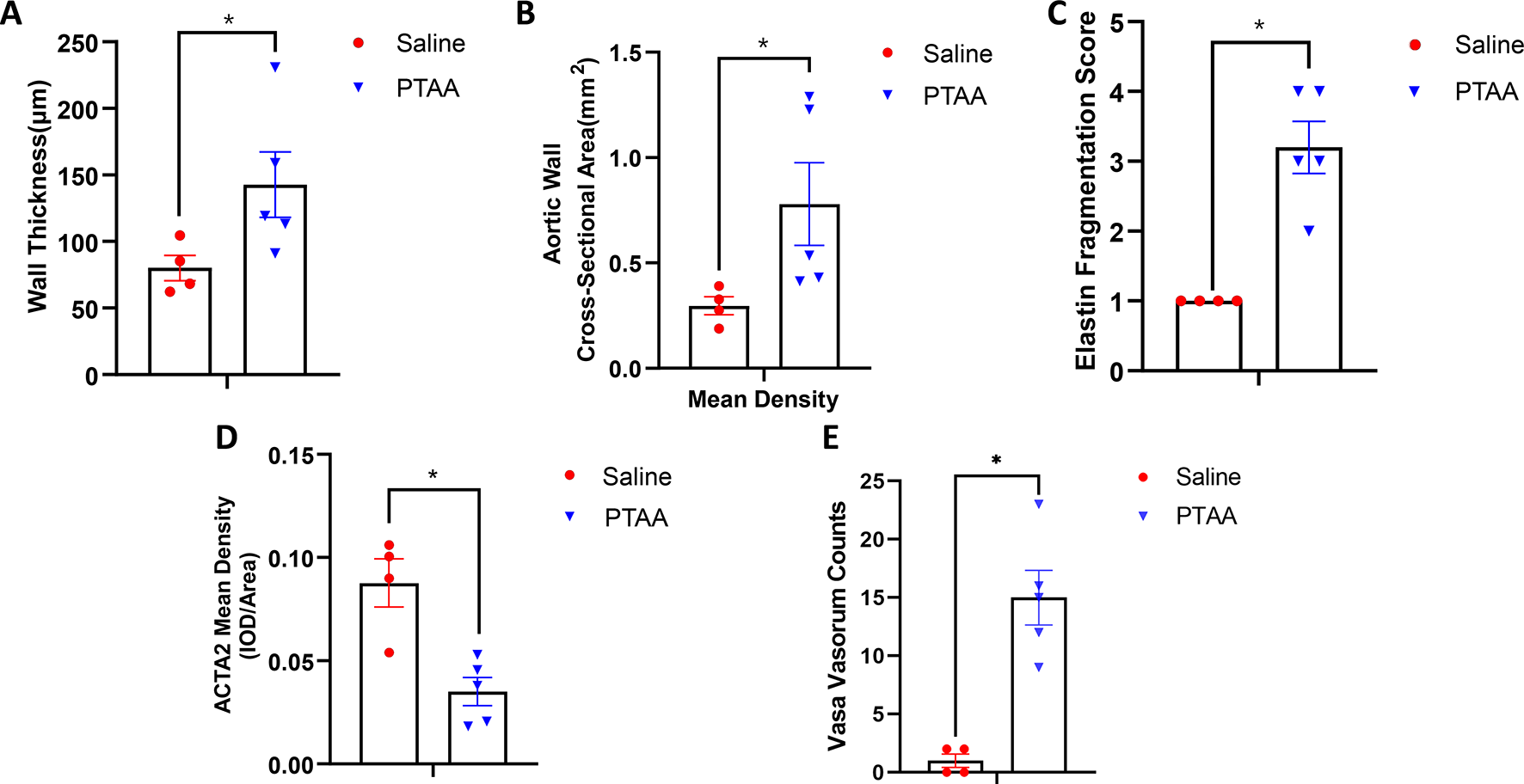

Supplement: Supplementary Figure 1 — Statistical quantative analysis of aortic wall thickness (A), cross-sectional area (B), the elastin fragmentation score (C), ACTA2 protein density (D), and the vasa vasorum counts (E) of the ascending aorta in the elastase application group and the saline group. Mean ± s.e.m; two-tailed Mann–Whitney tests, *P < 0.05. Elastin fragmentation was graded as follows: grade 1, intact, well-organized elastic laminae; grade 2, elastic laminae with some interruptions and breaks; grade 3, severe elastin fragmentation or loss; and grade 4, severe elastin degradation with visible ruptured sites. [file Image_1.TIF]
